# Supplementary figures and images for: Survival benefit of adjuvant therapy following neoadjuvant therapy in patients with resected esophageal cancer: A retrospective cohort study
Source: PLoS One. 2024 Nov 19;19(11):e0304937. doi: 10.1371/journal.pone.0304937 (PMC11575812; doi:10.1371/journal.pone.0304937)

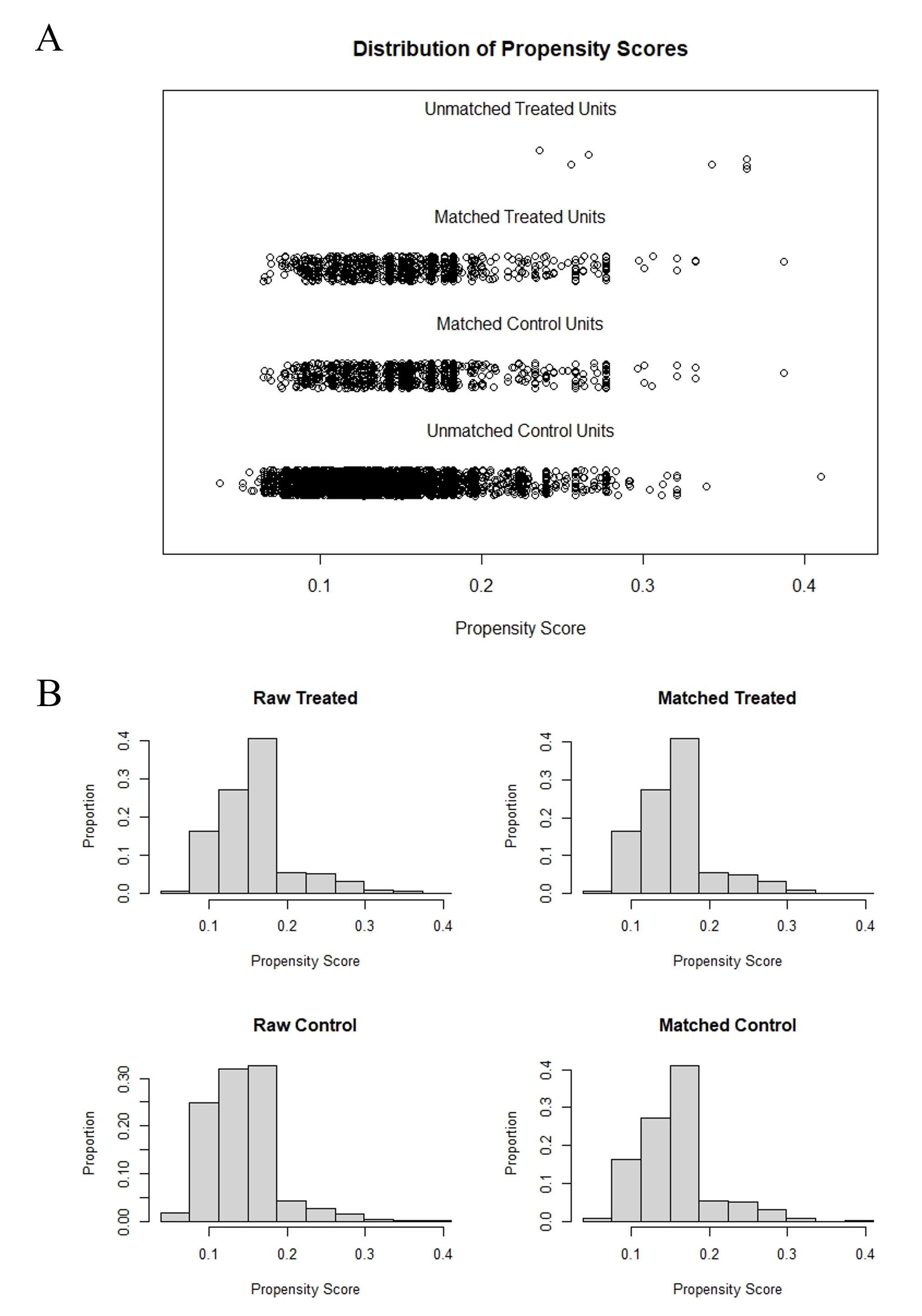

Supplement: S1 Fig — Distribution (A) and histograms (B) of propensity scores before and after propensity score matching. (TIF) [file pone.0304937.s001.tif]

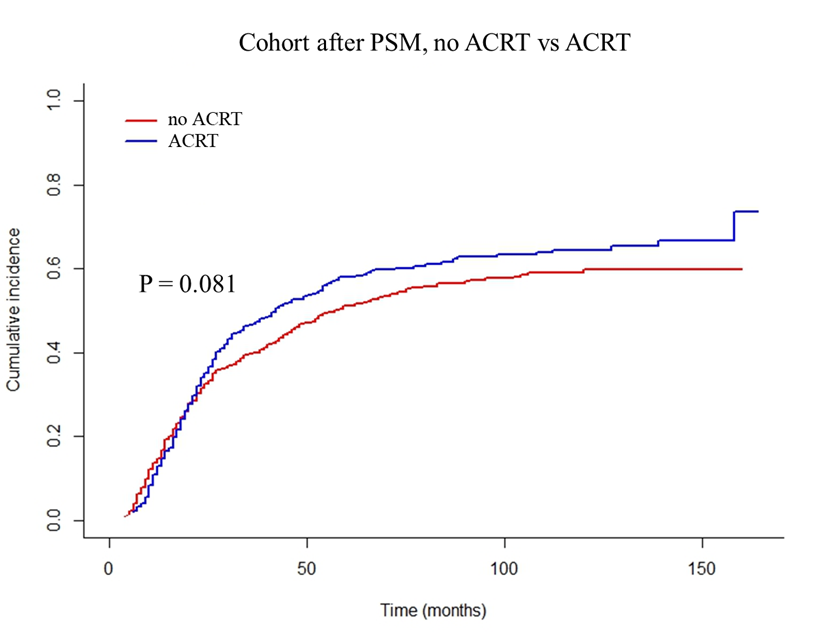

Supplement: S2 Fig — (TIF) [file pone.0304937.s002.tif]

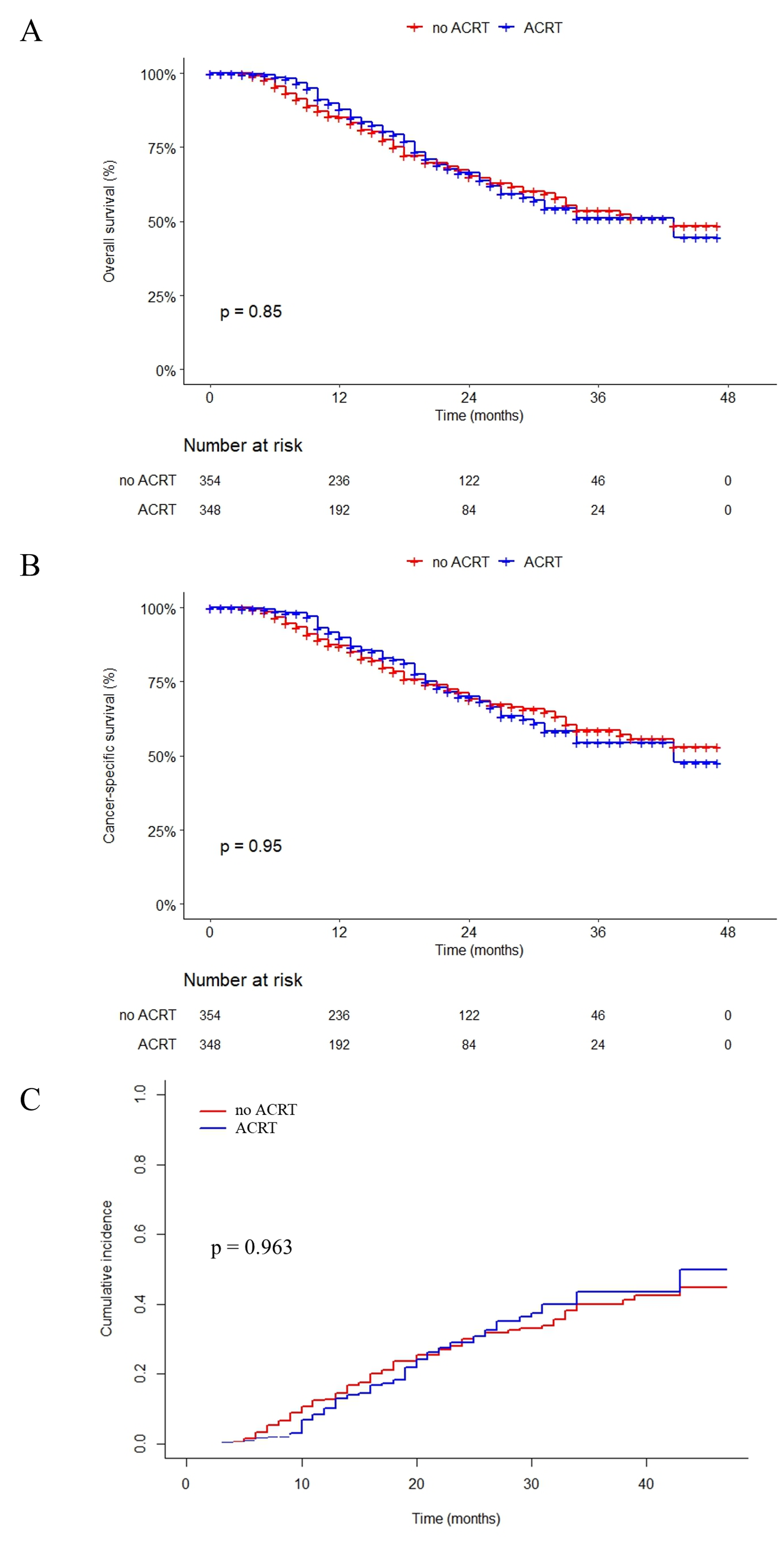

Supplement: S3 Fig — Kaplan-Meier curves for OS (A) and CSS (B). Cumulative incidence curve for CSS (C). ACRT, adjuvant chemotherapy or radiotherapy. (TIF) [file pone.0304937.s003.tif]
