# Supplementary material for: Survival benefit of adjuvant therapy following neoadjuvant therapy in patients with resected esophageal cancer: A retrospective cohort study
Source: PLoS One. 2024 Nov 19;19(11):e0304937. doi: 10.1371/journal.pone.0304937 (PMC11575812; doi:10.1371/journal.pone.0304937)
Supplement: S1 Table — (DOCX) [file pone.0304937.s004.docx]

Table S1. Univariate COX regression analysis for cancer-specific survival and overall survival.

| Variables | CSS | |  | OS | |
| --- | --- | --- | --- | --- | --- |
|  | HR (95% CI) | P |  | HR (95% CI) | P |
| Age |  |  |  |  |  |
| ≤ 65 | Ref | 0.247 |  | Ref | 0.005 |
| > 65 | 1.090 (0.942-1.261) |  |  | 1.212 (1.061-1.385) |  |
| Year of diagnosis |  |  |  |  |  |
| 2007-2011 | Ref | 0.039 |  | Ref | 0.035 |
| 2012-2016 | 0.872 (0.739-1.029) | 0.105 |  | 0.853 (0.732-0.995) | 0.042 |
| 2017-2020 | 0.778 (0.638-0.949) | 0.013 |  | 0.803 (0.668-0.967) | 0.020 |
| Gender |  |  |  |  |  |
| Female | Ref | 0.118 |  | Ref | 0.049 |
| Male | 1.211 (0.952-1.540) |  |  | 1.252 (1.001-1.566) |  |
| Race |  |  |  |  |  |
| Other | Ref | 0.467 |  | Ref | 0.187 |
| White | 1.094 (0.767-1.559) | 0.621 |  | 1.183 (0.844-1.660) | 0.330 |
| Black | 1.365 (0.813-2.291) | 0.239 |  | 1.554 (0.962-2.510) | 0.072 |
| Marital |  |  |  |  |  |
| Other | Ref | 0.082 |  | Ref | 0.135 |
| Married | 0.825 (0.679-1.002) | 0.052 |  | 0.859 (0.716-1.029) | 0.099 |
| Single | 0.965 (0.743-1.253) | 0.789 |  | 0.989 (0.776-1.262) | 0.931 |
| T classification |  |  |  |  |  |
| T1 | Ref | <0.001 |  | Ref | 0.002 |
| T2 | 0.941 (0.697-1.272) | 0.694 |  | 0.856 (0.657-1.116) | 0.251 |
| T3 | 1.348 (1.064-1.709) | 0.014 |  | 1.186 (0.966-1.456) | 0.104 |
| T4 | 1.767 (1.221-2.555) | 0.003 |  | 1.451 (1.035-2.034) | 0.031 |
| N classification |  |  |  |  |  |
| N0 | Ref | <0.001 |  | Ref | <0.001 |
| N1 | 1.434 (1.203-1.709) | <0.001 |  | 1.272 (1.088-1.486) | <0.001 |
| N2 | 1.540 (1.152-2.060) | 0.004 |  | 1.390 (1.054-1.833) | 0.003 |
| N3 | 3.346 (2.054-5.451) | <0.001 |  | 1.789 (1.059-3.023) | <0.001 |
| TNM stage |  |  |  |  |  |
| I | Ref | <0.001 |  | Ref | <0.001 |
| II | 1.114 (0.801-1.550) | 0.520 |  | 0.982 (0.739-1.306) | 0.903 |
| III | 1.650 (1.208-2.253) | 0.002 |  | 1.412 (1.080-1.847) | 0.012 |
| Histology |  |  |  |  |  |
| ADC | Ref | 0.505 |  | Ref | 0.372 |
| SCC | 0.925 (0.742-1.153) | 0.487 |  | 0.900 (0.733-1.103) | 0.310 |
| Other | 1.099 (0.880-1.374) | 0.405 |  | 1.090 (0.888-1.337) | 0.411 |
| Differentiation |  |  |  |  |  |
| Well | Ref | <0.001 |  | Ref | <0.001 |
| Moderately | 0.823 (0.569-1.192) | 0.303 |  | 0.864 (0.613-1.217) | 0.403 |
| Poorly | 1.104 (0.770-1.583) | 0.589 |  | 1.132 (0.810-1.582) | 0.467 |
| Unknown | 0.726 (0.480-1.099) | 0.130 |  | 0.735 (0.501-1.080) | 0.117 |
| ACRT after surgery |  |  |  |  |  |
| No | Ref | 0.154 |  | Ref | 0.883 |
| Yes | 1.111 (0.962-1.283) |  |  | 1.010 (0.885-1.153) |  |
| CSS, cancer-specific survival; OS, overall survival; Ref, reference; HR, hazard ratio; CI, confidence interval; ADC, adenocarcinoma; SCC, squamous cell carcinoma; ACRT, adjuvant chemotherapy or radiotherapy. | | | | | |
